# Pathogens infecting the central nervous system

**DOI:** 10.1371/journal.ppat.1010234

**Published:** 2022-02-24

**Authors:** Yohann Le Govic, Baptiste Demey, Julien Cassereau, Yong-Sun Bahn, Nicolas Papon

**Affiliations:** 1 Infectious Agents, Resistance and Chemotherapy (AGIR), University of Picardy Jules Verne, Amiens, France; 2 Parasitology-Mycology Department, Center for Human Biology, University Hospital of Amiens-Picardie, Amiens, France; 3 Virology Department, Center for Human Biology, University Hospital of Amiens-Picardie, Amiens, France; 4 Department of Neurology, Angers University Hospital, Angers, France; 5 Univ Angers, Inserm, CNRS, MITOVASC, SFR ICAT, Angers, France; 6 Department of Biotechnology, College of Life Science and Biotechnology, Yonsei University, Seoul, Republic of Korea; 7 Univ Angers, Univ Brest, IRF, SFR ICAT, Angers, France; University of Maryland, Baltimore, UNITED STATES

## Introduction

**Fig 1:**
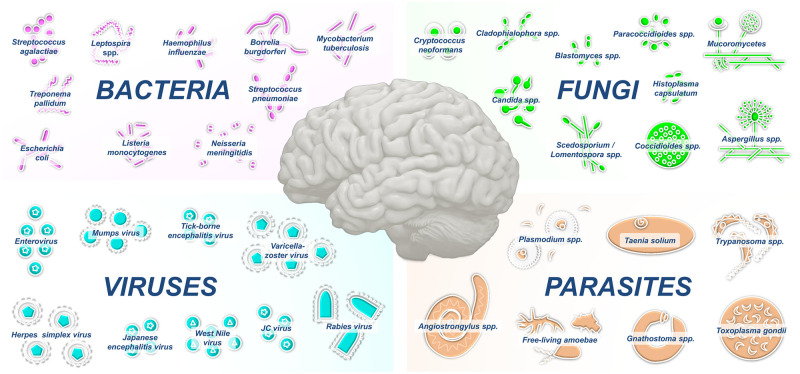
Compilation of prominent bacteria, fungi, viruses, and parasites that infect the CNS. CNS, central nervous system.

Infections of the central nervous system (CNS) are among the most devastating infectious diseases worldwide and often result in medical emergencies that require prompt management. Pathogens may access the CNS by crossing the blood–brain barrier (BBB), which normally protects the CNS from microbial invasion, or via transneuronal routes that bypass the BBB. A broad array of infectious agents can cause CNS infections in the meningeal or parenchymal compartments (Fig 1, Table 1). Infection of the cerebrospinal fluid (CSF) and its surrounding meninges, termed meningitis, is accompanied with the acute onset of fever, headache, and neck stiffness. Infection of the CNS parenchyma leads to encephalitis, which clinically involves fever, neuropsychological impairment, and seizures. By contrast, CNS infection confined to small areas of focal lesions or abscesses are more likely to occur in immunocompromised individuals. Here, we summarize the etiologies of these potentially vaccine-preventable infections, their transmission routes, and the recent advances in understanding the mechanisms of CNS invasion by different neurotropic pathogens.

**Table 1:** Etiology and epidemiology of CNS infections.

Pathogen	Geographic distribution	Transmission route	Demographics	Clinical presentation
Bacteria				
*Streptococcus agalactiae*	Worldwide	Vertical transmission (mother to child) through the birth canal	Neonates	Meningitis
*Escherichia coli*				
*Neisseria meningitidis*		Inhalation (droplets produced by coughing or sneezing)	Children and adults	
*Streptococcus pneumoniae*				
*Haemophilus influenzae*			Elderly and immunocompromised individuals	
*Listeria monocytogenes*		Transplacental	Elderly, immunocompromised, (neonates)	Meningitis, rhombencephalitis
*Mycobacterium tuberculosis*	Worldwide; vast majority in Africa/Asia	Inhalation (Flugge droplets)	Children and adults Immunocompromised (HIV infected patients)	Meningitis, cerebral tuberculomas
*Treponema pallidum* (neurosyphilis)	Worldwide	Direct contact (sexual)	Adults	Meningoencephalitis; General paresis; Tabes dorsalis
*Leptospira* spp.	Worldwide	Contact with infected mammals (rodents)	Children and adults	Meningitis; Meningoencephalitis; Myelitis
*Borrelia burgdorferi* (neuroborreliosis)	North America and Eurasia	Arthropod borne (tick: *Ixodes* spp.)	Children and adults	Encephalitis; Meningitis; Encephalopathy
Viruses				
EV	Worldwide	Fecal/oral Inhalation (EV-D68)	Children and adults	Meningitis Encephalitis (rare)
HSV	Worldwide	Skin/mucosa	Children (mainly HSV1) and adults (mainly HSV2) Immunocompromised	Meningitis Encephalitis
VZV	Worldwide	Skin/mucosa Inhalation	Adults (mostly immunocompromised)	Encephalitis; Meningitis (rare); Myelitis
Mumps virus	Worldwide	Inhalation	Children and adults (mostly unvaccinated)	Meningitis; Myelitis;
Rabies virus	Worldwide; vast majority in Africa/Asia	Contact with infected mammal (dogs)	Children and adults	Encephalitis
WNV	Worldwide	Arthropod borne (mosquito: *Culex* spp.)	Children and adults (mostly elderly population)	Meningitis; Encephalitis
JEV	Asia, Australia, and western Pacific	Arthropod borne (mosquito: *Culex* spp.)	Children and adults (mostly pediatric population)	Encephalitis
TBEV	Central and northern Europe	Arthropod borne (tick: *Ixodes ricinus*)	Children and adults	Encephalitis
JCV	Worldwide	Inhalation	Adults with severe immune deficiency	PML
Fungi				
*Candida* spp. (neurocandidiasis)	Worldwide (human commensal)	Nosocomial (neurosurgery, CNS devices)	Preterm neonates, children, and adults	Meningoencephalitis; brain abscesses
*Cryptococcus neoformans* (neurocryptococcosis)	Worldwide; frequent in Europe	Inhalation (bird droppings)	Immunocompromised (especially for CD4^+^ T-cell counts <100/mm^3^)	Meningoencephalitis
*Aspergillus* spp.	Worldwide	Inhalation	Immunocompromised	Brain abscesses (frequently secondary to lung infections)
*Scedosporium* spp. *Lomentospora* spp.	Worldwide	Inhalation	Children and adults (mostly immunocompromised)	Brain abscesses (secondary to lung infections or near-drowning)
Mucoromycetes (*Rhizopus*, *Lichtheimia*, *Mucor* …)	Worldwide	Inhalation	Immunocompromised	Brain abscesses (frequently secondary to sinus infections)
*Histoplasma capsulatum* (histoplasmosis)	Central and eastern United States (var. capsulatum); Africa (var. duboisii)	Inhalation (bird or bat droppings)	Children and adults (immunocompetent)	Meningitis, meningoencephalitis, and brain abscesses
*Blastomyces* spp. (blastomycosis)	North America	Inhalation (soil dust)	Children and adults (immunocompetent)	Meningitis, meningoencephalitis, and brain abscesses
*Coccidioides* spp. (coccidioidomycosis)	Southwest US, Mexico, and South America	Inhalation (soil dust)	Children and adults (immunocompetent)	Meningitis, meningoencephalitis, and brain abscesses
*Paracoccidioides* spp. (paracoccidioidomycosis)	Central and South America	Inhalation (soil dust)	Children and adults (immunocompetent)	Brain abscesses and meningitis
Dematiaceous molds (phaeohyphomycosis)	Worldwide	Inhalation	Children and adults (immunocompetent)	Brain abscesses
Parasites				
*Toxoplasma gondii*	Worldwide	Ingestion of tissue cysts or sporulated oocysts Organ transplant Blood transfusion	Immunocompromised (especially for CD4^+^ T-cell counts <200/mm^3^)	Encephalitis and brain abscesses
*Trypanosoma brucei* (sleeping disease)	Africa	Arthropod borne (Tsetse flies, Glossinidae)	Children and adults	Mental and behavioral disorders, sleep and sleep–wake cycles disturbances
*Plasmodium* spp. (cerebral malaria)	Tropical and subtropical regions	Arthropod borne (Mosquito: *Anopheles* spp.)	Children and adults	Impaired consciousness and coma
*Taenia solium* (neurocysticercosis)	Africa, Asia, and Latin America	Fecal–oral	Children and adults (mostly from resource-limited countries)	Intracerebral cysts (epilepsy)
*Angiostrongylus* spp. (neuroangiostrongyliasis)	Southeast Asia, Oceania, and the Americas	Ingestion of terrestrial mollusks (snails/slugs)	Children and adults	Eosinophilic meningitis; Intracranial hemorrhage
*Gnathostoma* spp. (gnathostomiasis)	Southeast Asia, Japan Korea, and Latin America	Food borne (mostly raw freshwater fish, amphibians, and reptiles)	Children and adults	Eosinophilic meningitis; Intracranial hemorrhage
Free-living amoebae (*Acanthamoeba*, *Balamuthia* and *Naegleria*)	Worldwide	Inhalation (contaminated water)	Immunocompromised (*Acanthamoeba*, *Balamuthia*) Immunocompetent (*Naegleria*)	Granulomatous amebic encephalitis (*Acanthamoeba*, *Balamuthia*) Primary amebic meningoencephalitis (*Naegleria*)

CNS, central nervous system; EV, enterovirus; HSV, herpes simplex virus; JCV, JC virus; JEV, Japanese encephalitis virus; PML, progressive multifocal leukoencephalopathy; TBEV, tick-borne encephalitis virus; VZV, varicella-zoster virus; WNV, West Nile virus.

### Bacterial infections

The CNS may be infected by a wide variety of bacteria (Fig 1, Table 1). The spectrum of these infections varies from focal infections, such as brain abscesses, to generalized entities such as meningoencephalitis. Contiguous spread from the upper airways, hematogenous spread from another primary site, and direct inoculation through trauma or surgery can contribute to the development of a CNS bacterial infection. The etiology of bacterial meningitis varies according to age group and immune status. The most frequent infective agents affecting newborns in the first week, *Streptococcus agalactiae* and *Escherichia coli*, are replaced by *Streptococcus pneumoniae* and *Neisseria meningitidis* by the sixth week [1]. Subsequently, *S*. *pneumoniae* remains the most common bacterial agent, followed by *N*. *meningitidis* and *Listeria monocytogenes* [1,2]. *Haemophilus influenzae* type B (Hib) used to be a leading cause of pediatric meningitis in the pre-Hib vaccination era [3]. Similarly, the introduction of pneumococcal and meningococcal conjugate vaccines has substantially reduced the burden of bacterial meningitis. Gram-negative bacilli and *Staphylococcus* spp. are the most common causes of nosocomial CNS infections.

Atypical bacteria can also reach the brain. *Mycobacterium tuberculosis* causes tuberculous meningitis, which originates from a pulmonary focus that spreads via the lymphatic system but also intracranial tuberculomas. The local inflammatory response leads to nerve palsy and alterations in the CSF and cerebral blood flow. The mortality rate in treated cases remains high, ranging from 20% to 67% [4]. Spirochetes are also responsible for CNS diseases that are mediated by local inflammatory responses. *Treponema pallidum*, a sexually transmitted pathogen, usually cause neurosyphilis in the late stage of infection [5]. Pathogenic *Leptospira* spp. cause leptospirosis, an acute febrile disease mainly transmitted by brown rats, eliciting neurological complications or meningoencephalitis [6]. *Borrelia burgdorferi*, an agent of a tick-borne Lyme borreliosis, causes encephalitis or meningitis associated with arthropathies [7].

### Viral infections

Viral meningitis is by far the most frequent clinical presentation of CNS infections (Fig 1, Table 1). Enteroviruses (EVs), members of the Picornaviridae family, are involved in approximately 90% of cases [8]. EV-A71, EV-D68, and Coxsackievirus B are most frequently detected in patients with aseptic meningitis. Human parechoviruses (HPeVs), especially HPeV-3, are other Picornaviridae that are commonly responsible for meningitis. EVs can replicate in the upper respiratory and intestinal epithelial cells and then disseminate into the bloodstream and CNS through infected immune cells [9]. EV meningitis is usually a benign, self-limiting condition.

The Herpesviridae family is the second leading cause of viral meningitis. Herpes simplex virus 2 (HSV-2) is the predominant causal agent, but HSV-1, varicella-zoster virus (VZV), and Epstein–Barr virus (EBV) also cause meningitis. HSV-2 meningitis may occur as a result of primary infection or reactivation [10]. Mumps virus, causing parotitis and hearing loss, is one of the main causes of viral meningitis in unvaccinated populations [11].

In addition to meningitis, most of the aforementioned viruses are also capable of causing encephalitis. HSV-1 can cause necrotic acute encephalitis during reactivation in the adult population. Similarly, VZV encephalitis is common after reactivation of the virus (zoster). Other viruses are typically characterized as causal agents of encephalitis. Historically, rabies is the most popular viral brain infection and caused by various *Lyssavirus* species that are transmitted by dog bites in approximately 99% of human cases [12]. Animal control and vaccination programs aim to prevent infections that occur in approximately 150 countries and affect approximately 3 billion people.

Arthropod-borne viruses (arboviruses) are responsible for encephalitis in endemic areas. Over the years, however, climatic and ecological changes have altered the geographical distribution of arboviral infections. The West Nile virus (WNV) rapidly emerged in Northern America and Southern Europe during the 21st century. This *Flavivirus* is transmitted by mosquitoes and often cause encephalitis especially in elderly patients. The Japanese encephalitis virus (JEV) is another mosquito-borne *Flavivirus* and causes viral encephalitis in Asia. Tick-borne encephalitis virus (TBEV) is the third most common encephalitis-causing arbovirus. This *Flavivirus* is primarily transmitted to humans by the widespread hard tick species *Ixodes ricinus* [13]. Although the mortality rate is low, up to 30% of patients with TBEV encephalitis develop neurological sequelae. CNS diseases may be caused by other neurotropic viruses such as human immunodeficiency virus (HIV), cytomegalovirus, human herpesvirus 6, influenza virus, or measles virus [12].

Finally, JC virus (JCV), a polyomavirus that commonly establishes asymptomatic infection in the general population, is responsible for progressive multifocal leukoencephalopathy (PML), a fatal demyelinating disease of the CNS, in patients with severe immune deficiency. The development of new immunomodulatory and immunosuppressive drugs expanded the spectrum of conditions associated with PML [14].

### Fungal infections

Unlike bacteria, fungi are eukaryotic (mostly saprophytic) organisms with membrane-bound nuclei that obtain nutrients from organic matter. Fungal infections of the CNS are commonly opportunistic, resulting from hematogenous dissemination in immunocompromised hosts. However, immunocompetent individuals are increasingly being reported as possible hosts for such infections. The fungal infections often originate from direct inoculation (e.g., trauma or surgery) of fungal spores.

Medically important fungi that invade the CNS include yeasts, molds (filamentous fungi), and dimorphic fungi (Fig 1, Table 1). CNS-infecting yeasts include a number of ubiquitous species, such as *Candida* spp. and *Cryptococcus neoformans*, the latter showing strong neurotropism. CNS-infecting molds include hyalohyphomycetes with septate hyphae (e.g., *Aspergillus* and *Scedosporium/Lomentospora* species) and the mucormycetes with nonseptate or sparsely septate hyphae (e.g., *Mucor*, *Rhizopus*, and *Lichtheimia* species). These fungi are distributed worldwide. Phaeohyphomycetes (dark molds) represent a third group of ubiquitous neurotropic molds (e.g., *Cladophialophora bantiana*, *Exophiala dermatitidis*, and *Rhinocladiella mackenziei*). Dimorphic fungi, such as *Histoplasma*, *Blastomyces*, *Coccidioides*, and *Paracoccidioides*, have a confined geographical distribution in the American continents and often infect the CNS [15].

Importantly, the morphology of the fungus influences the pathogenesis of CNS lesions. Fungi that develop as budding yeasts in vivo primarily cause meningitis (dimorphic fungi) or meningoencephalitis (*Cryptococcus* and *Candida* species). Those that exhibit yeast-to-hyphae transition (e.g., *Candida albicans*) can be more invasive, leading to necrosis and brain abscesses. Those that grow large hyphae (i.e., filamentous fungi) have a propensity for macrovascular invasion, causing hemorrhagic stroke, aneurysms, and cerebral abscesses. Cryptococcal meningoencephalitis is the most frequent fungal infection of the CNS, whereas candidiasis is the most common nosocomial infection. Aspergillosis and mucormycosis are relatively rare but devastating in immunosuppressed patients, while cerebral phaeohyphomycoses mainly occur in immunocompetent individuals [16]. Besides immunological disorders, some environmental, iatrogenic, and host-related factors may predispose an individual to the fungal CNS infection.

### Parasitic infections

Parasitic diseases involving the CNS are major threats, especially in low- and middle-income countries. The causative agents include miscellaneous unicellular and multicellular organisms such as protozoa and worms, respectively (Fig 1, Table 1). Certain parasitic agents are highly dreaded in specific contexts, such as cerebral malaria in travelers returning from endemic regions with fever and any neurological symptoms or cerebral toxoplasmosis in patients infected with HIV. Parasitic infections of the CNS may be suspected in patients with nonspecific manifestations, such as meningitis, encephalitis, ventriculitis, myelitis, or brain abscess, with fever and headaches as chief complaints. The clinical presentation depends on the localization and size of the lesions, but distinct parasites may lead to the same symptomatology, making diagnosis challenging. Although a number of CNS parasitic infections are endemic in tropical countries, they are now spreading globally due to international migration and travel [17].

Nematode infections are the main cause of eosinophilic meningoencephalitis (especially *Angiostrongylus* and *Gnathostoma* species). In addition, neurocysticercosis, caused by larval cysts of the tapeworm *Taenia solium*, is the most common cause of epileptic seizures in low-income countries. In this respect, extraparenchymal forms (i.e., outside the brain tissue) result in high morbidity and mortality. In a pathophysiological perspective, the inflammatory response toward the larva is the hallmark of the disease and is supposed to contribute to BBB breakdown. Some protozoan species are also known for infecting the human CNS. This is notably the case of the flagellate *Trypanosoma brucei*, the etiological agent of African trypanosomiasis (sleeping sickness), which induces life-threatening meningoencephalitis. Although much rarer, the pathogenic free-living amoebae (*Acanthamoeba*, *Balamuthia*, and *Naegleria* species) are noteworthy causal agents due to their high case-fatality rates.

### BBB crossing mechanisms of CNS-infecting pathogens

The human BBB is a neurovascular unit composed of brain microvascular endothelial cells (BMECs), pericytes, astrocytic end feet, microglia, and neurons. The presence of tight junctions (TJs) and adherens junctions (AJs) makes paracellular movements of even a small molecule extremely difficult [18]. Nevertheless, neurotropic pathogens can cross the BBB via (i) transcellular migration; (ii) paracellular migration; and/or (iii) a Trojan horse mechanism. In the transcellular mechanism, a pathogen binds to BMECs, then is taken up by BMECs through receptor-mediated endocytosis, is transported within a vacuole without fusion with lysosomes, and is finally released to the brain tissues. In the paracellular mechanism, a pathogen can traverse between BMECs by disrupting TJs and/or AJs, which can be facilitated by the induced expression of pro-inflammatory cytokines, such as tumor necrosis factor alpha (TNFα), interleukin (IL)-1β, IL-6, and interferon gamma (IFNγ), in BMECs, pericytes, and astrocytes. In the Trojan horse mechanism, phagocytes infected with a pathogen cross the BBB paracellularly.

Several CNS-infecting bacteria, including *E*. *coli*, group B *Streptococcus*, *S*. *pneumoniae*, *N*. *meningitidis*, *L*. *monocytogenes*, and *M*. *tuberculosis*, cross the BBB transcellularly (see [19,20]). The latter 2 pathogens also cross the BBB via a Trojan horse mechanism. Bacterial surface adhesins are generally required for BBB crossing. These include CD48-interacting type I fimbrial adhesin FimH and gp96-interacting outer-membrane protein A in *E*. *coli*, a platelet-activating factor receptor-interacting cell wall phosphorylcholine in *S*. *pneumoniae*, laminin-binding protein, the fibrinogen-binding protein FbsA and invasion-associated gene A in *S*. *agalactiae*, and type IV pili PilC in *N*. *meningitidis*. In addition, several invasion proteins modulate host cytoskeleton regulation to promote transcellular traversal of bacterial pathogens.

Viruses can infect the CNS by either directly traversing the BBB through one of the mechanisms described above or by taking nonhematogenous routes, including retrograde axonal transport from peripheral nerves to the CNS and the nasal olfactory epithelium and neurons. In particular, CNS-infecting viruses can stimulate the production of pro-inflammatory cytokines and matrix metalloproteases in BMECs, astrocytes, and pericytes, which can destabilize TJs by activating the RhoA kinase pathway and promoting BBB crossing of neurotropic viruses [21,22].

Among the various neuroinfectious fungal pathogens, 2 pathogenic yeasts, *C*. *albicans* and *C*. *neoformans*, traverse the BBB transcellularly [23]. Inositol, which is abundantly present in the brain, is taken up by *C*. *neoformans* through inositol transporters Itr1a and Itr3c and induces the expression of hyaluronic acid (HA) synthase gene *CPS1* in *C*. *neoformans*. The HA produced enhances the binding of the fungal pathogen to the CD44 glycoprotein in blood endothelial cells [24,25]. *C*. *neoformans*–derived extracellular microvesicles and the metalloprotease Mpr1 also contribute to the BBB crossing process of *C*. *neoformans* [26,27]. Recent systematic BBB crossing analyses of signature-tagged mutants and CRISPR/Cas-9–based gene deletion mutants in *C*. *neoformans* revealed that a variety of proteins involved in diverse biological functions are involved in BBB crossing and pathogen survival in the brain parenchyma [28]. In addition, *C*. *neoformans* can cross the BBB via Trojan horse mechanism [29]. *C*. *albicans* can cross the BBB by utilizing fungal invasins, Als3 and Ssa1, the former of which can interact with the gp96 receptor on BMECs [30].

The BBB crossing mechanisms of parasites are relatively well studied in *Toxoplasma gondii* and trypanosomes [31]. *T*. *brucei* can cross the BBB by expressing the cysteine protease cathepsin L (brucipain) that interacts with G-protein coupled receptors on host endothelial cells. *T*. *gondii* can cross the BBB in a Trojan horse mechanism. In this process, *T*. *gondii* secretes cyclophilin 18, which interacts with the chemokine receptor CCR5 present on phagocytic cells.

### Toward new therapeutic approaches for treating CNS infections

Therapeutic options for CNS infections are highly limited, because the delivery of antimicrobial agents to the affected brain compartments is challenged by the structural complexity and tightness of the human BBB. To study the molecular mechanisms of microbial CNS invasion and expedite screening drugs for CNS infections and disorders, intensive efforts have been made to construct in vitro BBB models in the past years. The simplest but most widely used in vitro BBB model is a transwell system containing a monolayer of human BMECs with or without astrocytes [32]. Microfluidic devices have recently been developed to better reflect complex three-dimensional BBB structures [33]. Most recently, the human neurovascular unit (hNVU) chip, which contains all the necessary cellular and extracellular brain components, was used to examine the neurotropism and BBB penetration of *C*. *neoformans* [34]. Although screening and development of antimicrobial agents with a good BBB permeability are important, the application of BBB-penetrating conjugates could be a promising approach. In particular, a number of peptide-based BBB shuttles have been developed in the past decades [35–36]. These BBB shuttles could be applied to a variety of currently available antimicrobial agents in future.

In conclusion, recent advances in ex/in vivo and in vitro BBB and CNS models will not only facilitate understanding of the CNS infection pathophysiology, but also support the screening of novel antimicrobial agents for the treatment of microbial meningitis. Better treatment strategy targeting CNS infections is an essential prerequisite to improving the global management of these life-threatening microbial infections.
